# Non-interpersonal traumatic events in patients with eating disorders: a systematic review

**DOI:** 10.3389/fpsyg.2024.1397952

**Published:** 2024-06-17

**Authors:** Kirstine Marie Johnsen, Kirstine Franciska Nielsen, Kristine Kahr Nilsson, Gry Kjaersdam Telléus

**Affiliations:** ^1^Institute of Communication and Psychology, Psychology, Aalborg University, Aalborg, Denmark; ^2^Psychiatry, Aalborg University Hospital, Aalborg, Denmark

**Keywords:** trauma, non-interpersonal, eating disorder, anorexia nervosa, bulimia nervosa

## Abstract

**Objectives:**

The purpose of the systematic review was to synthesize literature on eating disorders (ED) and non-interpersonal traumatic events (NTE) and consolidate the reported prevalence of NTE in patients with an ED.

**Methods:**

The literature search was performed in Embase, PsycInfo, and PubMed. The keywords in the search were “eating disorder,” “trauma” and “non-interpersonal,” using index-terms and free-search keywords related to NTE and ED. The PRISMA guidelines were followed. Relevant studies were screened using Rayyan.

**Results:**

Of the 16 studies included in the quantitative synthesis, five overall types of NTE were identified: *accidents*, *illness*, *injury*, *natural disaster* and *war*. Findings provided tentative evidence for *illness* and *injury* being more prevalent in patients suffering from an ED compared to controls. The remaining subtypes of NTE did not show a higher prevalence in patients with an ED when compared to controls. Findings also suggest that those with binge/purge subtype of anorexia nervosa (AN) had a higher prevalence of non-interpersonal traumatic events compared to the restrictive subtype of AN.

**Discussion:**

This systematic review provided a clear synthesis of previous findings related to NTE among patients with an ED. Noteworthy, is that many studies do not take into account if the trauma happened prior or after to ED onset, which may affect the association. Furthermore, the body of research on NTE in patients with ED is exceedingly limited, and more research is needed.

## Introduction

Trauma involves a wide variety of potentially stressful exposures traditionally divided into two types of trauma, namely, interpersonal trauma (e.g., the direct result of actions by others, such as abuse and neglect), and non-interpersonal trauma (e.g., other life-threatening events, such as accident, injury, illness, war, and natural disaster; Forbes et al., 2011; Hughesdon et al., 2021; Thomas et al., 2021).

Highly stressful and traumatic events are regarded as potential precipitating factors for the onset of ED, and studies indicate that individuals with an ED are more prone to report a history of trauma (Garner and Garfinkel, 1980; Meneguzzo et al., 2021; Sundhedsstyrelsen, 2021; Rienecke et al., 2022). This was also highlighted in the systematic review by Rossi et al. (2024) on the concept of the maltreated eco-phenotype of ED, exploring the distinctive biological and clinical features associated with childhood maltreatment (CM). Rossi et al. (2024) synthesized evidence revealing significant neuroanatomical changes, stress response alterations, inflammation markers, and gut microbiota composition differences among individuals with EDs who had experienced CM. Importantly, these findings stress the significance of viewing CM and interpersonal trauma not only as risk factors but also as causal agents shaping various eco-phenotypic variants of EDs. The comprehensive nature of this review provides a valuable framework for understanding the complex interplay between trauma and the development of EDs, an association that may also apply to other types of trauma, such as NTE. Prior research investigating NTE’s association with psychopathology found that NTE had a significant association with PTSD, anxiety, and depressive symptoms (Haldane and Nickerson, 2016; Smith et al., 2023). This raises the question of whether there is also evidence of an association between NTE and ED across studies. The majority of studies examining the link between trauma and ED tend to focus on interpersonal traumas as observed by Rossi et al. (2024), thus leaving NTE relatively understudied (Trottier and MacDonald, 2017). The primary domains of interpersonal trauma that have been investigated in the literature of ED, are physical-, emotional-, and sexual abuse (Forman-Hoffman et al., 2012; Kothari et al., 2015; Trottier and MacDonald, 2017). Two meta-analyses have found that these interpersonal forms of traumatic events were associated with increased ED pathology (Caslini et al., 2016; Molendijk et al., 2017). Thus, Caslini et al. (2016) found a noteworthy association between distinct types of childhood abuse and ED subtypes, but it was discovered that only BN and BED demonstrated distinct connections with all forms of child abuse (Caslini et al., 2016). Molendijk et al. (2017) showed overall consistent results with the meta-analyses of Caslini et al. (2016). However, in addition, Molendijk et al. (2017) found a high prevalence of child abuse in each type of ED, AN included (Molendijk et al., 2017). Overall, the literature suggests that the binge/purge subtypes of EDs are frequently associated with different types of interpersonal traumatic events. Beyond the association observed between interpersonal trauma and AN, BN, and BED, more recent studies have also uncovered a link between interpersonal trauma and the ED subtypes OSFED and ARFID (Scharff et al., 2021; Rienecke et al., 2022). Nevertheless, few studies have examined the relationship between NTE and different ED subtypes. Moreover, the investigation of NTE in patients with an ED possesses clinical importance because trauma exposure has been identified as a complicating factor in the treatment of ED, leading to increased rates of dropout and ED pathology post-treatment (Convertino and Mendoza, 2023; Day et al., 2024). Research has shown that individuals with an ED who have experienced a traumatic event exhibit heightened severity of ED pathology, elevated levels of anxiety and depressive symptoms, and lower levels of mindfulness compared to those who have not experienced trauma (Rabito-Alcon et al., 2021; Scharff et al., 2021). Hence, it is imperative to investigate the prevalence of NTE in patients with ED to determine the necessity of incorporating this trauma type into ED treatment strategies. Additionally, it has not been investigated, which subtypes of NTE have the highest prevalence among patients with ED. Regarding interpersonal traumas, the traumas with a bodily dimension, such as sexual and physical abuse are often found to have a high prevalence in patients with ED (Caslini et al., 2016). Therefore, we predict that non-interpersonal traumas with a bodily dimension such as *illness* and *injury* have the highest prevalence in patients with ED. Given this knowledge gap in the literature concerning NTE in the context of ED, it is deemed relevant to investigate the prevalence of NTE in patients with ED in a systematic review.

## Objectives and aim

This systematic review aimed to investigate the prevalence of NTE in patients diagnosed with ED.

Based on existing literature on the association between interpersonal traumatic events and ED, the following hypotheses were proposed:

1. Individuals with an ED are more likely to have been exposed to a NTE
2. The prevalence of NTE varies across different subtypes of ED with a higher prevalence in the binge/purge subtypes
3. The NTE subtypes of *injury* and *illness* have are more prevalent than other NTEs in individuals with ED.

Literature regarding traumatic events and ED describes traumatic events as a triggering factor (Garner and Garfinkel, 1980). Therefore, if data allows it, we wish to investigate the temporal frame by the following hypothesis.

1. The traumatic event happened prior to ED onset.

## Method

### Search strategy

The systematic review followed the *2020 Preferred Reporting Items for Systematic Reviews and Meta-Analyses* (PRISMA) standard (Page et al., 2021). Additionally, the systematic review protocol was structured in accordance with the PRISMA-P guidelines. A literature search was carried out by the chief librarian of Aalborg University Hospital, Denmark on 17 October 2023, in the databases PubMed, PsycInfo, and Embase. No time restrictions were imposed, and all identified relevant articles found in the databases were included in the systematic review. The search phases combined controlled vocabularies (e.g., Mesh, thesaurus, and Emtree) and free-search terms relevant to the Population (individuals with an ED), Comparison (presence or absence of ED), and Outcome (prevalence of NTE) components of the PICO framework. Besides these keywords, other reviews were excluded by filters in PubMed and Embase in the search. A comprehensive list of the search terms, synonyms, and controlled vocabularies, used in the different databases can be found in Supplementary Table 1. The extraction files were then uploaded into Rayyan:^1^ an online reference management software. After removing duplicates by Rayyan’s duplicate-identification strategies the articles were screened for relevance, language, and availability. All searches, starting from the initial screening of the title and abstract to the final decision on inclusion through full-text reading, were conducted independently by two reviewers (K.M.J., K.F.N.). To reduce the risk of errors made in the selection of studies, the reviewers were blinded to each other. Discrepancies between the two reviewers were resolved through consensus. If persistent disagreements arose, a senior researcher (G.K.T.) was consulted as a third party to reach a final decision. This approach ensured a rigorous screening process and minimized the potential for bias in study selection.

### Inclusion and exclusion criteria

All studies collected from the literature search were screened based on the predetermined inclusion and exclusion criteria. To be included in the systematic review, studies had to be peer-reviewed and have a quantitative research design. Additionally, the prevalence had to be extractable or calculable from the articles’ data. Participants in the studies were required to have or have had a clinical diagnosis of ED according to the *International Classification of Diseases* (ICD-10) or the *Diagnostic and Statistical Manual of Mental Disorders* (DSM-IV and DSM-5), to be included. Studies on comorbid disorders and medical conditions were not excluded from the systematic review. This decision stemmed from the well-established overlap between EDs, and diverse medical and psychiatric conditions documented in existing literature (Hudson et al., 2007). By incorporating individuals with comorbidity, a more nuanced depiction of the complex presentation of EDs was achieved. Additionally, the studies had to include NTE among participants with a current or previous ED diagnosis. Inclusion criteria specified that studies had to have a sample size of 10 or more individuals diagnosed with an ED.

Studies that only reported an overall category of trauma were excluded, as NTE could not be differentiated from interpersonal traumatic events. Studies were also excluded if they had other research designs than empirical study designs. Moreover, studies in languages other than Danish, English, Swedish, or Norwegian were excluded.

### Data extraction

In the data collection process, the following information was extracted from the included studies: (a) author names, (b) year of publication, (c) country, (e) population frame (e.g., in-patients, out-patients, etc.), (f) the different ED subcategories and assessment tools, (g) trauma type and measurement, (h) main findings regarding prevalence among individuals with and without ED. This form was used to summarize and synthesize the overall findings in relation to the objectives of the systematic review. The primary outcome of interest of the included studies was prevalence. If data were raw or numerical, the percentage was calculated. Only the relevant control groups for the objectives of this study were stated in the form. Throughout the article, we employed the term ‘control group’. This designation encompassed both reference groups as well as matched control groups without ED. We contacted the corresponding authors by mail if the prevalence of NTE could not be extracted from the articles but was expected to be in the possession of the authors. Articles were excluded if no response was received.

### Quality assessment

All included studies were assessed using the *Joanna Briggs Institute* (JBI) *Critical Appraisal Checklist for Studies Reporting Prevalence Data,* to critically evaluate the methodological quality of the included studies (Munn et al., 2020). This tool was used to evaluate different items in the studies, with responses categorized as “Yes,” “No,” “Unclear,” or “Not applicable.” JBI checklist was applied as it offered tailored evaluation criteria for prevalence studies, ensuring a thorough assessment of methodological quality in the included studies in a systematic and standardized way. With inspiration from Rasmussen et al. (2023), the total score for the JBI checklist for prevalence studies ranged from 1 to 9. The overall evaluation of the quality of the studies clarified the impact of the studies.

## Results

### Study selection

**Figure 1:**
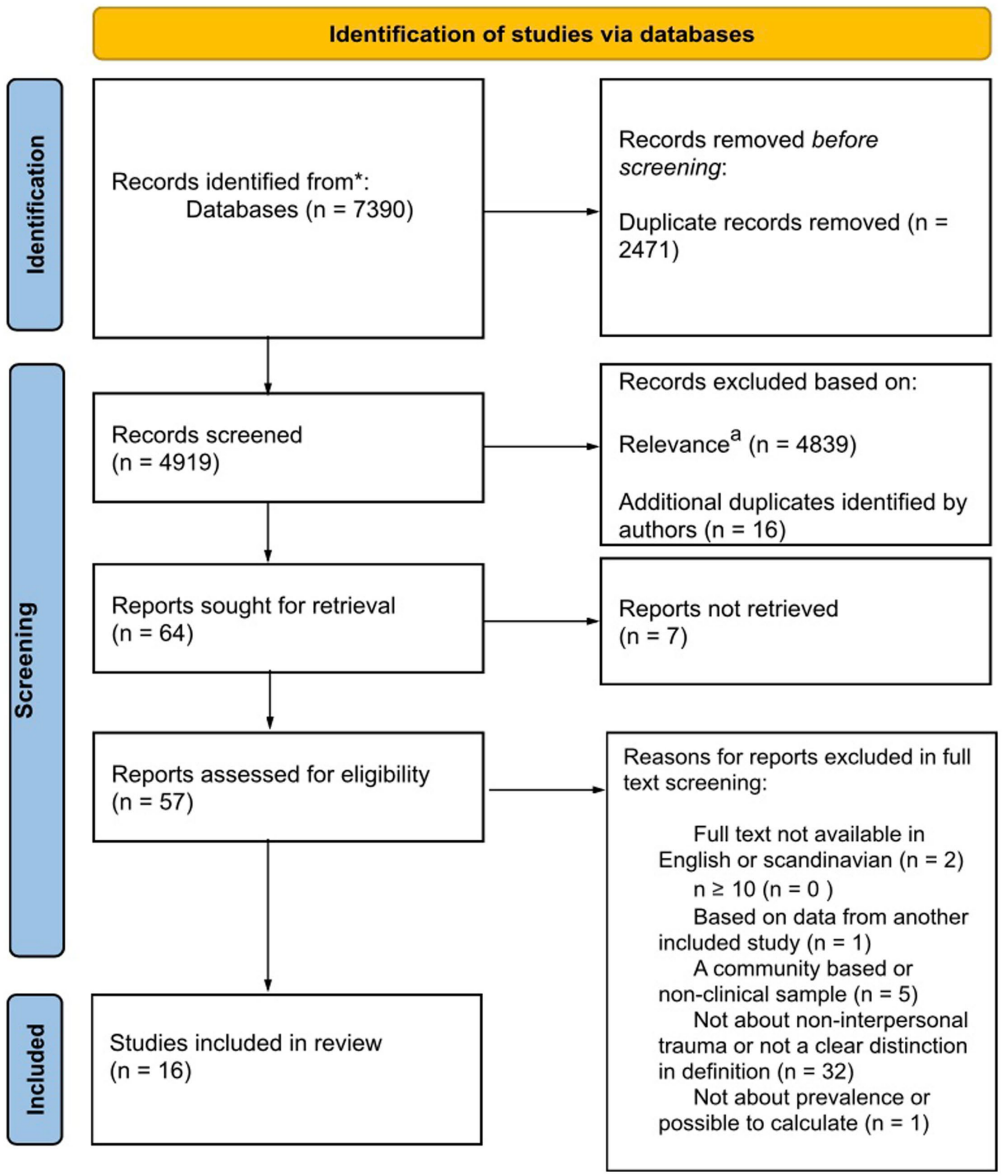
^a^Relevance in the initial screening was determined by assessing whether the articles screened met the inclusion and exclusion criteria outlined in their titles and abstracts. *PubMed, Embase and PsycInfo.

A total of 7,390 articles were retrieved in the initial search. From these, 2,624 came from PubMed, 3,160 from Embase, and 1,606 from PsycInfo. Five articles were identified under the subheading PsycTest that were excluded in the initial search process. Only references under the subheadings PsycInfo and PsycArticles were included. The removal of duplicates resulted in 2471 articles, and another 16 duplicates were subsequently removed. The following screening by title and abstract resulted in 64 articles, of which seven articles could not be retrieved. The agreement rate between authors in the independent screening was 97.9%. 57 articles were assessed according to the predetermined exclusion and inclusion criteria, of which 16 were included. Figure 1 depicts a detailed description of the study selection in a flow diagram.

### Study characteristics

Supplementary Table 2 shows data from the 16 included research articles. The number of participants in the ED group ranged from *n* = 21 (Thornley et al., 2016) to *n* = 4,524 (Backholm et al., 2013). For the control groups included in the studies, the sample sizes ranged from *n* = 245 (Lejonclou et al., 2014) to *n* = 35,709 (Convertino et al., 2022). Thirty-seven-point 5 % of the studies involved only adult participants (>18 years; Reyes-Rodríguez et al., 2011; Backholm et al., 2013; Tagay et al., 2014; Thornley et al., 2016; Longo et al., 2019; Brewerton et al., 2020) while 6.25% included child/adolescent participants (<18 years; Groth et al., 2020). When including Kjaersdam Telléus et al. (2021), who also investigated adolescence, but in the age range from 15 to 19.5, the percentage increased to 12.5%. Half of the studies either included both age groups or did not clarify the age range (Tagay et al., 2010; Degortes et al., 2014; Lejonclou et al., 2014; Vieira et al., 2018; White et al., 2018; Longo et al., 2020; Lie et al., 2021; Convertino et al., 2022). Results indicated a varied prevalence of NTE in the child/adolescent and adult age groups, with no consistent trend observed between the two groups.

The included studies predominantly originated from the United States and Europe, with minimal to no representation from other regions such as Asia or Africa. Of the included studies six and four studies investigated only out- and inpatients, respectively. When comparing the two groups, inpatients exhibited a higher prevalence of *illness* or *injury* ranging from 20 to 59% (Thornley et al., 2016; Longo et al., 2019; Brewerton et al., 2020; Longo et al., 2020), while outpatients showed a range of 6.5–19.4%, when Lejonclou et al. (2014) study was excluded from the outpatient range due to significantly higher results (e.g., 60% had been in the hospital and 70% had experienced having a family member in the hospital; Tagay et al., 2010; Degortes et al., 2014; Vieira et al., 2018; White et al., 2018; Convertino et al., 2022).

In general, different measurements were used to measure eating pathology and exposure to traumatic events, including both structured clinical interviews and self-report questionnaires. Supplementary Table 3 provides a list of all the measurements used in the studies. Overall, 31% of the studies used the *Life Events Checklist* (LEC) to assess the prevalence of traumatic events in patients with an ED (Backholm et al., 2013; Thornley et al., 2016; Longo et al., 2019; Brewerton et al., 2020; Longo et al., 2020).

Despite using the same measurement instrument, LEC, no consistency was observed in the prevalence of NTE subtypes across the five studies, but rather a significant variation ranging from 0–59%. For instance, in Backholm et al. (2013), only 1.57% of patients with ED had experienced life-threatening *illness* or *injury*, whereas Thornley et al. (2016) reported a much higher prevalence of 43% for this variable.

### NTE experienced by patients with ED

Two of the 16 studies presented the prevalence of an overall category of NTE in patients suffering from ED. In these, the prevalence was, respectively, 75.2% (Convertino et al., 2022) and 14.3% (Longo et al., 2020). Across the 16 studies, NTE could be grouped under five broad subheadings: *accidents*, *injury*, *illness*, *war*, and *natural disaster*. Overall, the prevalence of the different NTEs in patients with an ED across all included studies ranged from 0 to 72%. The results were ambiguous, but generally, the prevalence was between 0 and 30%. The studies that showed the highest prevalence were Brewerton et al. (2020), Lejonclou et al. (2014), Tagay et al. (2014) and Thornley et al. (2016). Generally, *illness* and *injury* showed the highest prevalence compared to the other subtypes of NTE (Backholm et al., 2013; Degortes et al., 2014; Lejonclou et al., 2014; Tagay et al., 2014; Thornley et al., 2016; Longo et al., 2019; Convertino et al., 2022). When differentiating these, *illness* had the highest prevalence of the two variables (Convertino et al., 2022). In contrast to these findings, Tagay et al. (2010) and Brewerton et al. (2020) found that *accidents* were the subtype with the highest prevalence. However, most of the studies showed that *accidents* had a lower prevalence than *illness* and *injury*, but a higher prevalence than *natural disasters* and *war* (Reyes-Rodríguez et al., 2011; White et al., 2018).

### Patients with ED vs. control group

The studies that incorporated a control group were conducted by Convertino et al. (2022), Lejonclou et al. (2014), Lie et al. (2021), and Thornley et al. (2016). The included control groups used as a reference consisted of populations pooled from internet samples (Thornley et al., 2016; Lie et al., 2021), national surveys regarding alcohol and related conditions (Convertino et al., 2022), and recruitment from Secondary Schools and High Schools (Lejonclou et al., 2014). The clinical group was predominantly females, whereas gender was mixed in the control groups. Most of the studies had a control group that was representative of the clinical group in terms of age. However, Thornley et al. (2016) exhibited a greater difference.

Only one study reported the prevalence of the overall category of NTE in comparison to a control group (Convertino et al., 2022). In this study, the overall prevalence of NTE in patients suffering from ED was 75.2%, whereas the prevalence in the group without ED was 62.2%. Across studies with a control group, the prevalence of the subtypes of NTE in the ED group ranged from 0 to 70%, in comparison to 0.18–72% in the control group without ED. Two studies demonstrated a generally higher prevalence of *accidents* in the control group compared to the ED group (Thornley et al., 2016; Lie et al., 2021). The findings in Lejonclou et al. (2014) presented varied results regarding the prevalence of multiple items related to *accidents*.

**Figure 2:**
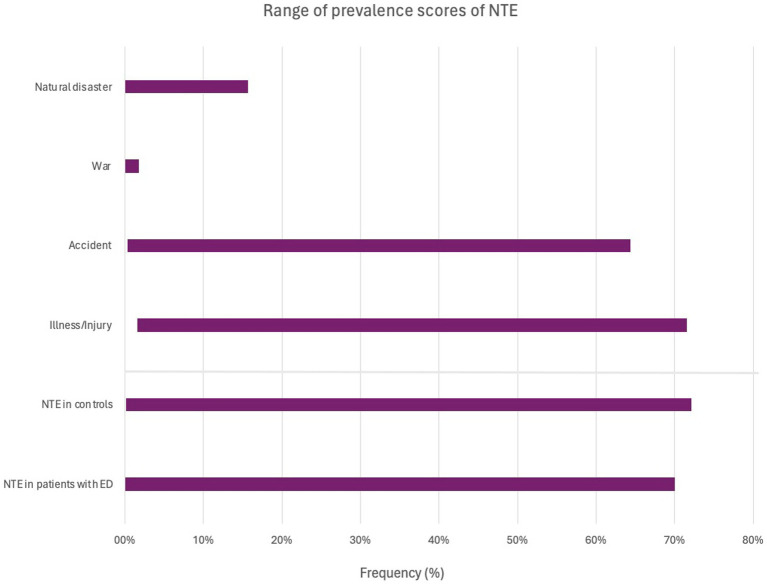
Graphical representation showing the range (maximum and minimum) of prevalence across NTE categories. It is important to note that the range is influenced by the varying numbers of studies and their sample sizes. The number of studies reporting the prevalence in regard to War, Natural disaster, Accident, lllness/injury, was 5, 9, 11, 12, respectively. The bottom part of the diagram shows the overall prevalence of NTE among patients with ED as indicated in studies with a control group, and the prevalence of NTE in these control groups. The number of studies with a control group was 4.

In all studies the prevalence of war exposure was more than twice as high in the control group, which indicated that the prevalence was considerably lower in the ED sample (Lejonclou et al., 2014; Thornley et al., 2016; Convertino et al., 2022). Likewise, ED groups demonstrated lower levels of exposure to *natural disasters* compared to control groups (Lejonclou et al., 2014; Thornley et al., 2016). In contrast, Convertino et al. (2022) reported that the rates of *natural disaster* did not differ between those with and without ED. The two variables *injury* and *illness* were sometimes combined in the measurement instruments for exposure to NTE (Thornley et al., 2016). Other studies separated the categories or only reported one of them (Lie et al., 2021; Convertino et al., 2022). The trauma measurement used in Lejonclou et al. (2014) included hospitalization as an item, which made it unclear whether it belonged to the category of *injury* or *illness*. Generally, the ED groups demonstrated a considerably higher prevalence of *illness* compared to the control groups (Lejonclou et al., 2014; Thornley et al., 2016; Lie et al., 2021; Convertino et al., 2022), although Lejonclou et al. (2014) showed no significant difference in the prevalence of *illness* when measuring hospitalization in a family member or close relative. In terms of *injury* there was not a significant difference between the ED- and control groups (Convertino et al., 2022). For a general overview of the main results regarding the minimum and maximum range for the prevalence of NTE, refer to Figure 2. Here, the difference in prevalence between the ED and control groups is also displayed within this interval range.

### Subtypes of ED

Most studies distinguished between the subtypes of ED: AN (Tagay et al., 2010; Reyes-Rodríguez et al., 2011; Lejonclou et al., 2014; Tagay et al., 2014; White et al., 2018; Longo et al., 2019; Groth et al., 2020; Longo et al., 2020; Kjaersdam Telléus et al., 2021; Lie et al., 2021; Convertino et al., 2022), BN (Tagay et al., 2010; Reyes-Rodríguez et al., 2011; Degortes et al., 2014; Lejonclou et al., 2014; Tagay et al., 2014; White et al., 2018; Groth et al., 2020; Kjaersdam Telléus et al., 2021; Lie et al., 2021; Convertino et al., 2022), BED (Degortes et al., 2014; Groth et al., 2020; Lie et al., 2021; Convertino et al., 2022) and OSFED (White et al., 2018; Groth et al., 2020; Kjaersdam Telléus et al., 2021). In terms of NTE reported in the single studies, there was not a large difference between AN, BN, BED, and OSFED. When the participants had the opportunity to report multiple lifetime ED diagnoses, it was shown that these participants (e.g., AN/BN and AN/BN/BED) had a higher prevalence than the participants with a single lifetime ED diagnosis (e.g., AN, BN or BED; Reyes-Rodríguez et al., 2011; Lie et al., 2021). The finding that the participants reporting multiple lifetime ED diagnoses, exhibits a higher prevalence of NTE compared to those with a single lifetime ED diagnosis, aligns with the findings of Rossi et al. (2024), which show an increased prevalence of the so-called diagnostic crossover in the maltreated eco-phenotype. The presence of diagnostic crossovers, such as individuals experiencing multiple forms of eating pathology over their lifetime, underscores the dynamic nature of EDs and emphasizes the need for a more nuanced understanding of ED that considers the interplay between trauma exposure, symptom expression, and diagnostic trajectories. Moreover, these findings underscore the importance of adopting a transdiagnostic approach clinically in assessment and treatment that accounts for the diverse pathways to ED development and maintenance.

It was demonstrated in Tagay et al. (2014), that participants with BN had experienced *accidents* and *illness*/*injury* to a higher extent compared to AN. However, other studies found that AN had the highest or same prevalence in relation to *accidents* and *illness*/*injury* (Tagay et al., 2010; Kjaersdam Telléus et al., 2021). When differentiating *illness* and *injury* it was shown in Convertino et al. (2022) that the highest prevalence of *injury* was seen in individuals with BED, in comparison to AN and BN. In contrast, BN was the ED subtype with the highest prevalence of *illness*. Only one study reported on *war* in relation to ED subtypes (Convertino et al., 2022). It was demonstrated that individuals with BN had been exposed to more war-related traumatic events, than AN and BED. However, the difference was minimal, and the percentage was very low. Findings showed that there was no consistent difference in the prevalence of *natural disasters* between AN- and BN subgroups in the included studies. ED subgroup comparisons showed that individuals with BED experienced fewer events related to *natural disaster*.

Regarding the difference between AN-R and AN-BP, it was demonstrated by Lie et al. (2021), Longo et al. (2019), and Kjaersdam Telléus et al. (2021), that individuals with AN-R had a lower prevalence of *accident* and *illness* than AN-BP. In terms of *natural disasters,* Longo et al. (2019) found that AN-R had the highest prevalence, in comparison to AN-BP. Similarly, Reyes-Rodríguez et al. (2011) showed a higher prevalence in individuals with AN-R compared to AN-P and AN-B in terms of *accidents*. However, this was not the case regarding *natural disaster*, where AN-P had the highest prevalence.

### Timing of NTE

Seven studies either exclusively reported the prevalence of NTE, when the events occurred before the onset of the ED diagnosis or provided information on the number of individuals who reported experiencing at least one NTE prior to the onset of the ED (Reyes-Rodríguez et al., 2011; Degortes et al., 2014; Tagay et al., 2014; Vieira et al., 2018; Longo et al., 2019, 2020; Lie et al., 2021). Between 58.3–81.1% of the ED sample reported the traumatic event happened before the ED onset. Noteworthy is that Tagay et al. (2014) and Reyes-Rodríguez et al. (2011), exclusively provided information on the timing of NTE in the subset of individuals with both ED and PTSD, omitting those who did not have PTSD. Additionally, two studies only investigated the prevalence of NTE occurring prior to the onset of ED (Degortes et al., 2014; Vieira et al., 2018).

### Quality of evidence

Thirteen of the 16 articles were assessed to have a quality that scored between four and six points out of nine, on the *Critical Appraisal Checklist for Studies Reporting Prevalence Data* in JBI. These articles were considered to have medium quality. Two studies achieved a score of eight, indicating a high quality. On the contrary, one of the included studies had a score of two, which is classified as low quality. Overall, the two high-quality studies showed a relatively low prevalence compared to the other studies, ranging from 0.15–14%.

## Discussion

The purpose of this systematic review was to investigate the prevalence of NTE in patients with ED through a quantitative overview of studies. Following a systematic literature search, 16 relevant articles were included in the systematic review. This is the first systematic review in the literature to investigate the prevalence of NTE in patients with ED.

Overall, the findings showed that there was a considerable variation in the reported prevalence of the different NTE in patients with ED in the included studies. There was evidence to suggest that *illness* was the NTE with the highest prevalence among patients with ED, whereas *war* and *natural disasters* had the lowest. Regarding this finding, *illness* was shown to be the only NTE subtype with a higher prevalence among patients with ED compared to controls. This is supported by the literature, which describes *physical illness* to be a significant risk factor for the development of an ED (Watkins et al., 2001; Goodwin et al., 2003). Additionally, the variable *illness* had a higher prevalence among individuals with ED compared to their respective control groups, as observed in Welch et al.’s (1997) study, which examined a community-based sample. This suggests that NTE with a bodily dimension has a stronger correlation with eating pathology, than other types of NTE. However, it is important to note that studies have shown that ED can prompt *physical illness* and *injury* such as bone fractures, osteoporosis, and heart diseases (Hall et al., 1989; Solmi et al., 2016). This could explain the elevated incidence of *illness* and *injury* among individuals with ED when the temporal frame is not taken into account. Beyond the explanation that individuals with ED may experience *illness* more frequently, there are indeed other hypotheses that could explain this association. One potential explanation involves the altered relationship with the body that individuals may develop following an illness. Prior studies have found that conditions like diabetes can profoundly affect one’s perception of and relationship with the body (Gemeay et al., 2015; Yagci et al., 2023). Additionally, the meta-analysis by Pinquart (2013) found that children and adolescents with chronic illnesses exhibited a less positive body image compared to a healthy control group. This altered relationship with the body could manifest in various ways, potentially contributing to the development or exacerbation of ED. Further investigation into these potential mechanisms could provide valuable insights into the complex interplay between *illness* and ED.

Significantly, half of the included studies failed to account for the timing of trauma, with nearly all utilizing a cross-sectional design. This aligns with existing literature highlighting the scarcity of longitudinal studies exploring trauma and ED (Trottier and MacDonald, 2017; Vanderlinden and Palmisano, 2018). The oversight regarding the timing of trauma impedes the establishment of a clear temporal relationship between trauma exposure and the onset of eating pathology, emphasizing the necessity of discussing causality. The absence of a temporal framework in these studies makes it difficult to determine the directionality of the observed association, as highlighted in the discussions by Kjaersdam Telléus et al. (2021) and Thomas et al. (2021). Consequently, it remains unclear whether trauma preceded the manifestation of ED symptoms or if the diagnosis of ED was prior to the traumatic event. Future research could benefit from incorporating prospective longitudinal study designs. Such an approach would facilitate a more nuanced exploration of the temporal framework, thereby enabling researchers to draw more informed conclusions about risk factors, causality, and maintenance factors (Trottier and MacDonald, 2017). By examining the timing of NTE in relation to ED symptoms, researchers may gain insights into the causal pathways and better elucidate the nature of the observed associations. Additionally, incorporating a causal perspective when assessing NTE could provide a more accurate description of the impact of traumatic experiences on the development and maintenance of ED.

The study by Thornley et al. (2016) did not explicitly state the temporal frame of the reported prevalence. Consequently, this study could not be included in the results section concerning the timing of NTE. However, Thornley et al. (2016) utilized the Perceived Causal Relations scale to assess the patient’s perception of a potential causal relationship between PTSD symptoms and the onset of the ED. Their findings suggested that the majority of patients with an ED perceived the development of the disorder to be influenced by PTSD symptoms.

According to prior literature, it can be assumed to be important to investigate when the trauma occurred in the individual’s life. This exploration is crucial for identifying critical periods and determining whether there are heightened rates of trauma in patients suffering from ED during specific life stages (Brewerton et al., 2022). In the included studies it was unclear whether exposure to a traumatic event mostly occurred in childhood, adolescence, or adulthood. The studies primarily investigated adults with only a few investigating children and/or adolescents. No difference was observed between these age groups. Vidaña et al. (2020) found in their study, that serious and life-threatening illness or injury and transportation accidents had a higher prevalence in individuals with disordered eating when these events occurred during childhood rather than adulthood. Conversely, the prevalence of *war* and *natural disasters* was higher in adulthood than in childhood. Most of the study participants were women and gender was not differentiated in the investigation of prevalence in the included studies. This complicated a generalization of the results to men with an ED. Previous studies have shown that gender has a moderating role in the psychological outcome of exposure to trauma (Cromer and Smyth, 2010; Haldane and Nickerson, 2016). The findings of the studies demonstrated that NTE in men was more strongly associated with psychological distress. In contrast, it is observed that interpersonal traumas had a greater impact on women. Gender differences regarding exposure to NTE and ED should therefore be considered in future research.

In relation to NTE in the various ED subtypes (e.g., AN, BN, BED, OSFED) the systematic review yielded mixed results. Nevertheless, the studies indicated that individuals with more than one ED diagnosis during their lifetime tended to exhibit a higher prevalence of NTE compared to those with a single ED diagnosis. In addition, we found that the association between NTE and ED was particularly strong for AN-BP, in comparison to AN-R. This aligns with previous studies that investigated other types of traumatic events (i.e., interpersonal traumatic events; Caslini et al., 2016; Molendijk et al., 2017; Palmisano et al., 2018; Rienecke et al., 2022). Additionally, the study by Longo et al. (2023) found that individuals with a history of childhood trauma and binging–purging behaviors, had a higher occurrence of dissociative symptoms, in comparison to those with restrictive eating behaviors.

Many of the studies excluded individuals with *Avoidant/Restrictive Food Intake Disorder* (ARFID) from their investigation, due to a small sample size. However, studies have suggested that ARFID is often a condition that emerges following a traumatic event that is attributed to eating (e.g., choking and vomiting; Kambanis et al., 2020; De Toro et al., 2021). Thus, incorporating ARFID into future research studies could provide insights into the prevalence of NTE in ARFID patients and enable comparison to the findings of the other ED subtypes.

Overall, the pooled data indicated that the prevalence of NTE ranged from 0 to 75.2%. In comparison to this, Molendijk et al. (2017) found that 21–59% of patients suffering from ED had been exposed to childhood maltreatment (CM), including emotional, physical, and sexual abuse. This is supported by a more recent study that found a prevalence between 45.2–62.9% (Eielsen et al., 2024). This association between ED and CM is well-established in the literature (Caslini et al., 2016; Quilliot et al., 2019; Rabito-Alcon et al., 2021; Chu et al., 2022). The higher prevalence of NTE among patients with a history of ED can be attributable to the higher prevalence of NTE in the general population compared to CM. Convertino et al. (2022) reported that the prevalence of any sexual interpersonal trauma, any other nonsexual interpersonal trauma, and any NTE in the non-ED group, were 9.1, 17.7, and 62.2%, respectively. Additionally, individuals with ED were found to be more likely to experience interpersonal traumatic events than NTEs compared to those without ED (Convertino et al., 2022). This is in line with studies comparing the association of NTE and interpersonal trauma to other types of psychopathologies (Forbes et al., 2011; Hughesdon et al., 2021; Thomas et al., 2021). Therefore, the association between NTE and ED may not be as robust as that observed between interpersonal traumas and ED. One possible explanation for this might be that interpersonal traumatic events involve a perpetrator, unlike NTE. Studies suggest that such involvement can result in feelings of betrayal of trust, a negative self-image, and a disruption of assumed beliefs about the external world (Tang et al., 2012; Martin et al., 2013; Zaccagnino et al., 2017). This aligns with the finding of Tagay et al. (2010), which indicated that interpersonal traumas were more frequently associated with severe PTSD symptoms than NTE. These underlying mechanisms involved in the association between the experience of traumatic events and ED, cannot be underlined in this systematic review.

Besides differentiating trauma types in NTE and interpersonal traumatic events, it could be beneficial to specify the duration of the trauma and the number of traumatic events, because the characteristics behind traumas which are acute, persistent, or repeated, are observed to be different (Kira, 2001; Littleton et al., 2007).

Among the studies included, there was noticeable diversity in how NTE was defined, leading to variations in the overall measurement of NTE. For instance, Convertino et al. (2022) did not consider *war* as an NTE. Additionally, there was variation in whether studies differentiated between *illness* and *injury* or combined them into one variable. In some assessment tools (e.g., LEC) being a witness to an event and personally experiencing an event were combined in the same prevalence report, while this combination did not occur in other measurement instruments. In future research, it could be beneficial to use a more narrowly focused research question, focusing on a single NTE (e.g., *illness* or *accidents*) or ED subtype (e.g., AN or BN), to enable the use of meta-analytical approaches. This would help to determine whether NTEs are more strongly related to some ED subtypes using statistical means.

When exploring the connection between ED and trauma, it is crucial to recognize that prevalence alone cannot stand as a determining factor. The experience of traumatic events varies among individuals, and personal factors such as psychosocial resources and biological vulnerabilities may contribute to determining whether an individual is traumatized by such events. Therefore, not everyone exposed to a potentially traumatic event exhibits PTSD symptoms (Wallick et al., 2022). Thus, it is important not only to document the prevalence of NTE but also to explore how the person has internalized the event and whether they qualify it as traumatic. To understand the association between the two, the focus should be on trauma-related symptoms rather than trauma exposure in future research.

### Strengths and limitations

The review was carried out following PRISMA guidelines for systematic reviews, which is in line with best practice. A strength of this systematic review was that the results were based on a large sample size of diagnosed ED subjects and controls in total. However, the sample size of ED in most of the included studies was small, which influenced the generalizability of their findings and consequently affected the quality of the systematic review. The systematic review sought to reduce bias using multiple reviewers in the screening- and extraction process, who were blinded to each other. This resulted in moderate to high interrater reliability. The review’s broad focus on ED diagnosis provided a comprehensive view of NTE in patients with ED, a topic not systematically reviewed previously. However, this breadth became a limitation due to high heterogeneity in study designs and measurement tools for assessing NTE and ED prevalence. This hindered comparison and prevented a reliable meta-analysis. Despite recognizing the potential benefits of a meta-analysis, the inherent data heterogeneity posed a significant barrier to its validity and reliability. Thus, we deliberately opted against a meta-analytic approach. Additionally, graphical representation of prevalence faced challenges due to inconsistent reporting standards and lack of confidence intervals in many studies, complicating aggregation, and visualization. The decision to exclude papers published in languages other than Danish, English, Swedish, or Norwegian was based on practical considerations. The researchers lacked proficiency in other languages, hindering their ability to assess the quality and relevance of papers accurately. While this approach may miss useful studies, it prioritizes the reliability, comprehensibility, and accessibility of the included literature.

### Implications in clinical practice

The results underline that patients with ED have not been exposed to NTE more than individuals without ED. NTE should not be viewed as a specific and high-risk factor in the development of ED. Thus, there should not be attached importance to NTE in the clinical work with ED. In comparison, interpersonal traumatic events are evaluated to have a higher impact on the occurrence of ED, which is why events such as sexual assaults and emotional violence should be assigned a higher priority in the assessment and treatment of ED.

## Conclusion

This study is, to the best of our knowledge, the first systematic review to investigate the prevalence of NTE in studies with clinical samples of individuals with ED. Overall, our results varied in prevalence across the studies but indicate that there was not a significant difference between patients with ED and controls except for the variables *illness* and *injury*. This suggests that individuals with ED have experienced *illness* and *injury* more often relative to the background population. *Illness* is also the NTE reported to have the highest prevalence of the five NTE subtypes: *accidents*, *injury*, *illness*, *natural disaster*, and *war*. No consistent difference was found between AN, BN, and BED regarding NTE. Although, AN-BP demonstrated a higher prevalence of NTE than AN-R, which aligns with the third hypothesis.

## Data availability statement

The original contributions presented in the study are included in the article/Supplementary material, further inquiries can be directed to the corresponding author.

## Author contributions

KJ: Writing – original draft. KFN: Writing – original draft. KKN: Writing – review & editing. GKT: Supervision, Writing – review & editing.

## Footnotes

^1^ http://rayyan.qcri.org
