# Supplementary material for: Non-interpersonal traumatic events in patients with eating disorders: a systematic review
Source: Front Psychol. 2024 Jun 17;15:1397952. doi: 10.3389/fpsyg.2024.1397952 (PMC11216314; doi:10.3389/fpsyg.2024.1397952)
Supplement: Supplementary file 1 [file Table_1.docx]

# **Supplementary material**

## **Table 1: Search Details in PubMed, Embase and PsycInfo**

## **PubMed 17.10.2023**

| Search number | Search Details | Results |
| --- | --- | --- |
| 9 | (("Feeding and Eating Disorders"[MeSH Terms] OR ("eating disorder*"[Title/Abstract] OR "anorexia*"[Title/Abstract] OR "binge eating*"[Title/Abstract] OR "bulimia"[Title/Abstract] OR "disordered eating"[Title/Abstract])) AND ("Accidents"[MeSH Terms] OR "Disasters"[MeSH Terms] OR "Disaster Victims"[MeSH Terms] OR "Warfare and Armed Conflicts"[MeSH Terms] OR ("non interpersonal*"[Title/Abstract] OR "noninterpersonal*"[Title/Abstract] OR "impersonal*"[Title/Abstract] OR "witness"[Title/Abstract] OR "bystander*"[Title/Abstract] OR "eyewitness"[Title/Abstract] OR "war"[Title/Abstract] OR "combat"[Title/Abstract] OR "injury*"[Title/Abstract] OR "injuries"[Title/Abstract] OR "fracture*"[Title/Abstract] OR "stressful event*"[Title/Abstract] OR "stressor*"[Title/Abstract] OR "trauma*"[Title/Abstract] OR "accident*"[Title/Abstract] OR "crash"[Title/Abstract] OR "collision*"[Title/Abstract] OR "unforeseen*"[Title/Abstract] OR "disaster*"[Title/Abstract] OR "fire"[Title/Abstract] OR "hurricane*"[Title/Abstract] OR "tsunami*"[Title/Abstract] OR "earthquake*"[Title/Abstract]))) NOT ("Review"[Publication Type] OR "Systematic Review"[Publication Type]) | 2,624 |
| 8 | "Review"[Publication Type] OR "Systematic Review"[Publication Type] | 3,338,309 |
| 7 | ("Feeding and Eating Disorders"[MeSH Terms] OR ("eating disorder*"[Title/Abstract] OR "anorexia*"[Title/Abstract] OR "binge eating*"[Title/Abstract] OR "bulimia"[Title/Abstract] OR "disordered eating"[Title/Abstract])) AND ("Accidents"[MeSH Terms] OR "Disasters"[MeSH Terms] OR "Disaster Victims"[MeSH Terms] OR "Warfare and Armed Conflicts"[MeSH Terms] OR ("non interpersonal*"[Title/Abstract] OR "noninterpersonal*"[Title/Abstract] OR "impersonal*"[Title/Abstract] OR "witness"[Title/Abstract] OR "bystander*"[Title/Abstract] OR "eyewitness"[Title/Abstract] OR "war"[Title/Abstract] OR "combat"[Title/Abstract] OR "injury*"[Title/Abstract] OR "injuries"[Title/Abstract] OR "fracture*"[Title/Abstract] OR "stressful event*"[Title/Abstract] OR "stressor*"[Title/Abstract] OR "trauma*"[Title/Abstract] OR "accident*"[Title/Abstract] OR "crash"[Title/Abstract] OR "collision*"[Title/Abstract] OR "unforeseen*"[Title/Abstract] OR "disaster*"[Title/Abstract] OR "fire"[Title/Abstract] OR "hurricane*"[Title/Abstract] OR "tsunami*"[Title/Abstract] OR "earthquake*"[Title/Abstract])) | 3,458 |
| 6 | "Accidents"[MeSH Terms] OR "Disasters"[MeSH Terms] OR "Disaster Victims"[MeSH Terms] OR "Warfare and Armed Conflicts"[MeSH Terms] OR "non interpersonal*"[Title/Abstract] OR "noninterpersonal*"[Title/Abstract] OR "impersonal*"[Title/Abstract] OR "witness"[Title/Abstract] OR "bystander*"[Title/Abstract] OR "eyewitness"[Title/Abstract] OR "war"[Title/Abstract] OR "combat"[Title/Abstract] OR "injury*"[Title/Abstract] OR "injuries"[Title/Abstract] OR "fracture*"[Title/Abstract] OR "stressful event*"[Title/Abstract] OR "stressor*"[Title/Abstract] OR "trauma*"[Title/Abstract] OR "accident*"[Title/Abstract] OR "crash"[Title/Abstract] OR "collision*"[Title/Abstract] OR "unforeseen*"[Title/Abstract] OR "disaster*"[Title/Abstract] OR "fire"[Title/Abstract] OR "hurricane*"[Title/Abstract] OR "tsunami*"[Title/Abstract] OR "earthquake*"[Title/Abstract] | 2,033,482 |
| 5 | "non interpersonal*"[Title/Abstract] OR "noninterpersonal*"[Title/Abstract] OR "impersonal*"[Title/Abstract] OR "witness"[Title/Abstract] OR "bystander*"[Title/Abstract] OR "eyewitness"[Title/Abstract] OR "war"[Title/Abstract] OR "combat"[Title/Abstract] OR "injury*"[Title/Abstract] OR "injuries"[Title/Abstract] OR "fracture*"[Title/Abstract] OR "stressful event*"[Title/Abstract] OR "stressor*"[Title/Abstract] OR "trauma*"[Title/Abstract] OR "accident*"[Title/Abstract] OR "crash"[Title/Abstract] OR "collision*"[Title/Abstract] OR "unforeseen*"[Title/Abstract] OR "disaster*"[Title/Abstract] OR "fire"[Title/Abstract] OR "hurricane*"[Title/Abstract] OR "tsunami*"[Title/Abstract] OR "earthquake*"[Title/Abstract] | 1,831,182 |
| 4 | "Accidents"[MeSH Terms] OR "Disasters"[MeSH Terms] OR "Disaster Victims"[MeSH Terms] OR "Warfare and Armed Conflicts"[MeSH Terms] | 349,051 |
| 3 | "Feeding and Eating Disorders"[MeSH Terms] OR "eating disorder*"[Title/Abstract] OR "anorexia*"[Title/Abstract] OR "binge eating*"[Title/Abstract] OR "bulimia"[Title/Abstract] OR "disordered eating"[Title/Abstract] | 67,952 |
| 2 | "eating disorder*"[Title/Abstract] OR "anorexia*"[Title/Abstract] OR "binge eating*"[Title/Abstract] OR "bulimia"[Title/Abstract] OR "disordered eating"[Title/Abstract] | 58,959 |
| 1 | "Feeding and Eating Disorders"[MeSH Terms] | 36,554 |

## **Embase 17.10.2023**

| No. | Query | Results |
| --- | --- | --- |
| #11 | ((('eating disorder'/exp OR ('eating disorder*':ti,ab,kw OR 'anorexia*':ti,ab,kw OR 'binge eating*':ti,ab,kw OR 'bulimia':ti,ab,kw OR 'disordered eating':ti,ab,kw)) AND (('accident'/exp OR 'disaster victim'/exp OR 'military phenomena'/exp) OR ('non interpersonal*':ti,ab,kw OR noninterpersonal*:ti,ab,kw OR impersonal*:ti,ab,kw OR witness:ti,ab,kw OR bystander*:ti,ab,kw OR eyewitness:ti,ab,kw OR war:ti,ab,kw OR combat:ti,ab,kw OR injury*:ti,ab,kw OR injuries:ti,ab,kw OR fracture*:ti,ab,kw OR 'stressful event*':ti,ab,kw OR stressor*:ti,ab,kw OR trauma*:ti,ab,kw OR accident*:ti,ab,kw OR crash:ti,ab,kw OR collision*:ti,ab,kw OR unforeseen*:ti,ab,kw OR disaster*:ti,ab,kw OR fire:ti,ab,kw OR hurricane*:ti,ab,kw OR tsunami*:ti,ab,kw OR earthquake*:ti,ab,kw))) NOT ('conference abstract'/it OR review/it)) AND [2000-2023]/py | 3160 |
| #10 | [2000-2023]/py | 29085583 |
| #9 | (('eating disorder'/exp OR ('eating disorder*':ti,ab,kw OR 'anorexia*':ti,ab,kw OR 'binge eating*':ti,ab,kw OR 'bulimia':ti,ab,kw OR 'disordered eating':ti,ab,kw)) AND (('accident'/exp OR 'disaster victim'/exp OR 'military phenomena'/exp) OR ('non interpersonal*':ti,ab,kw OR noninterpersonal*:ti,ab,kw OR impersonal*:ti,ab,kw OR witness:ti,ab,kw OR bystander*:ti,ab,kw OR eyewitness:ti,ab,kw OR war:ti,ab,kw OR combat:ti,ab,kw OR injury*:ti,ab,kw OR injuries:ti,ab,kw OR fracture*:ti,ab,kw OR 'stressful event*':ti,ab,kw OR stressor*:ti,ab,kw OR trauma*:ti,ab,kw OR accident*:ti,ab,kw OR crash:ti,ab,kw OR collision*:ti,ab,kw OR unforeseen*:ti,ab,kw OR disaster*:ti,ab,kw OR fire:ti,ab,kw OR hurricane*:ti,ab,kw OR tsunami*:ti,ab,kw OR earthquake*:ti,ab,kw))) NOT ('conference abstract'/it OR review/it) | 3639 |
| #8 | 'conference abstract'/it OR review/it | 8072963 |
| #7 | ('eating disorder'/exp OR ('eating disorder*':ti,ab,kw OR 'anorexia*':ti,ab,kw OR 'binge eating*':ti,ab,kw OR 'bulimia':ti,ab,kw OR 'disordered eating':ti,ab,kw)) AND (('accident'/exp OR 'disaster victim'/exp OR 'military phenomena'/exp) OR ('non interpersonal*':ti,ab,kw OR noninterpersonal*:ti,ab,kw OR impersonal*:ti,ab,kw OR witness:ti,ab,kw OR bystander*:ti,ab,kw OR eyewitness:ti,ab,kw OR war:ti,ab,kw OR combat:ti,ab,kw OR injury*:ti,ab,kw OR injuries:ti,ab,kw OR fracture*:ti,ab,kw OR 'stressful event*':ti,ab,kw OR stressor*:ti,ab,kw OR trauma*:ti,ab,kw OR accident*:ti,ab,kw OR crash:ti,ab,kw OR collision*:ti,ab,kw OR unforeseen*:ti,ab,kw OR disaster*:ti,ab,kw OR fire:ti,ab,kw OR hurricane*:ti,ab,kw OR tsunami*:ti,ab,kw OR earthquake*:ti,ab,kw)) | 6187 |
| #6 | ('accident'/exp OR 'disaster victim'/exp OR 'military phenomena'/exp) OR ('non interpersonal*':ti,ab,kw OR noninterpersonal*:ti,ab,kw OR impersonal*:ti,ab,kw OR witness:ti,ab,kw OR bystander*:ti,ab,kw OR eyewitness:ti,ab,kw OR war:ti,ab,kw OR combat:ti,ab,kw OR injury*:ti,ab,kw OR injuries:ti,ab,kw OR fracture*:ti,ab,kw OR 'stressful event*':ti,ab,kw OR stressor*:ti,ab,kw OR trauma*:ti,ab,kw OR accident*:ti,ab,kw OR crash:ti,ab,kw OR collision*:ti,ab,kw OR unforeseen*:ti,ab,kw OR disaster*:ti,ab,kw OR fire:ti,ab,kw OR hurricane*:ti,ab,kw OR tsunami*:ti,ab,kw OR earthquake*:ti,ab,kw) | 2487063 |
| #5 | 'non interpersonal*':ti,ab,kw OR noninterpersonal*:ti,ab,kw OR impersonal*:ti,ab,kw OR witness:ti,ab,kw OR bystander*:ti,ab,kw OR eyewitness:ti,ab,kw OR war:ti,ab,kw OR combat:ti,ab,kw OR injury*:ti,ab,kw OR injuries:ti,ab,kw OR fracture*:ti,ab,kw OR 'stressful event*':ti,ab,kw OR stressor*:ti,ab,kw OR trauma*:ti,ab,kw OR accident*:ti,ab,kw OR crash:ti,ab,kw OR collision*:ti,ab,kw OR unforeseen*:ti,ab,kw OR disaster*:ti,ab,kw OR fire:ti,ab,kw OR hurricane*:ti,ab,kw OR tsunami*:ti,ab,kw OR earthquake*:ti,ab,kw | 2347230 |
| #4 | 'accident'/exp OR 'disaster victim'/exp OR 'military phenomena'/exp | 331126 |
| #3 | 'eating disorder'/exp OR ('eating disorder*':ti,ab,kw OR 'anorexia*':ti,ab,kw OR 'binge eating*':ti,ab,kw OR 'bulimia':ti,ab,kw OR 'disordered eating':ti,ab,kw) | 102737 |
| #2 | 'eating disorder*':ti,ab,kw OR 'anorexia*':ti,ab,kw OR 'binge eating*':ti,ab,kw OR 'bulimia':ti,ab,kw OR 'disordered eating':ti,ab,kw | 82036 |
| #1 | 'eating disorder'/exp | 67434 |

## PsycInfo 17.10.2023

In total, 1,611 articles were identified on the search page, of which 5 were excluded because articles under the subheading: PsycTest wasn’t downloaded.

((((**IndexTermsFilt**: ("Accident Proneness")) *OR* (**IndexTermsFilt**: ("Falls")) *OR* (**IndexTermsFilt**: ("Home Accidents")) *OR* (**IndexTermsFilt**: ("Industrial Accidents")) *OR* (**IndexTermsFilt**: ("Pedestrian Accidents")) *OR* (**IndexTermsFilt**: ("Transportation Accidents")) *OR* (**IndexTermsFilt**: ("Disasters")) *OR* (**IndexTermsFilt**: ("Natural Disasters")) *OR* (**IndexTermsFilt**: ("War")) *OR* (**IndexTermsFilt**: ("Nuclear War")) *OR* (**IndexTermsFilt**: ("Combat Experience")))) *OR* (((**title**: ("non interpersonal*")) *OR* (**title**: (noninterpersonal*)) *OR* (**title**: (Impersonal*)) *OR* (**title**: (witness)) *OR* (**title**: (bystander*)) *OR* (**title**: (eyewitness)) *OR* (**title**: (war)) *OR* (**title**: (combat)) *OR* (**title**: (injury*)) *OR* (**title**: (injuries)) *OR* (**title**: (fracture*)) *OR* (**title**: ("stressful event*")) *OR* (**title**: (stressor*)) *OR* (**title**: (trauma*)) *OR* (**title**: (accident*)) *OR* (**title**: (crash)) *OR* (**title**: (collision*)) *OR* (**title**: (unforeseen*)) *OR* (**title**: (disaster*)) *OR* (**title**: (fire)) *OR* (**title**: (hurricane*)) *OR* (**title**: (tsunami*)) *OR* (**title**: (earthquake*))) *OR* ((**abstract**: ("non interpersonal*")) *OR* (**abstract**: (noninterpersonal*)) *OR* (**abstract**: (Impersonal*)) *OR* (**abstract**: (witness)) *OR* (**abstract**: (bystander*)) *OR* (**abstract**: (eyewitness)) *OR* (**abstract**: (war)) *OR* (**abstract**: (combat)) *OR* (**abstract**: (injury*)) *OR* (**abstract**: (injuries)) *OR* (**abstract**: (fracture*)) *OR* (**abstract**: ("stressful event*")) *OR* (**abstract**: (stressor*)) *OR* (**abstract**: (trauma*)) *OR* (**abstract**: (accident*)) *OR* (**abstract**: (crash)) *OR* (**abstract**: (collision*)) *OR* (**abstract**: (unforeseen*)) *OR* (**abstract**: (disaster*)) *OR* (**abstract**: (fire)) *OR* (**abstract**: (hurricane*)) *OR* (**abstract**: (tsunami*)) *OR* (**abstract**: (earthquake*))) *OR* ((**Keywords**: ("non interpersonal*")) *OR* (**Keywords**: (noninterpersonal*)) *OR* (**Keywords**: (Impersonal*)) *OR* (**Keywords**: (witness)) *OR* (**Keywords**: (bystander*)) *OR* (**Keywords**: (eyewitness)) *OR* (**Keywords**: (war)) *OR* (**Keywords**: (combat)) *OR* (**Keywords**: (injury*)) *OR* (**Keywords**: (injuries)) *OR* (**Keywords**: (fracture*)) *OR* (**Keywords**: ("stressful event*")) *OR* (**Keywords**: (stressor*)) *OR* (**Keywords**: (trauma*)) *OR* (**Keywords**: (accident*)) *OR* (**Keywords**: (crash)) *OR* (**Keywords**: (collision*)) *OR* (**Keywords**: (unforeseen*)) *OR* (**Keywords**: (disaster*)) *OR* (**Keywords**: (fire)) *OR* (**Keywords**: (hurricane*)) *OR* (**Keywords**: (tsunami*)) *OR* (**Keywords**: (earthquake*))))) *AND* ((((**IndexTermsFilt**: ("Eating Disorders")) *OR* (**IndexTermsFilt**: ("Anorexia Nervosa")) *OR* (**IndexTermsFilt**: ("Avoidant/Restrictive Food Intake Disorder")) *OR* (**IndexTermsFilt**: ("Binge Eating Disorder")) *OR* (**IndexTermsFilt**: ("Bulimia")) *OR* (**IndexTermsFilt**: ("Feeding Disorders")) *OR* (**IndexTermsFilt**: ("Hyperphagia")) *OR* (**IndexTermsFilt**: ("Kleine Levin Syndrome")) *OR* (**IndexTermsFilt**: ("Orthorexia")) *OR* (**IndexTermsFilt**: ("Pica")) *OR* (**IndexTermsFilt**: ("Purging (Eating Disorders)")) *OR* (**IndexTermsFilt**: ("Rumination (Eating)")))) *AND* (((**title**: ("eating disorder*")) *OR* (**title**: ("anorexia*")) *OR* (**title**: ("binge eating*")) *OR* (**title**: ("bulimia")) *OR* (**title**: ("disordered eating"))) *OR* ((**abstract**: ("eating disorder*")) *OR* (**abstract**: ("anorexia*")) *OR* (**abstract**: ("binge eating*")) *OR* (**abstract**: ("bulimia")) *OR* (**abstract**: ("disordered eating"))) *OR* ((**Keywords**: ("eating disorder*")) *OR* (**Keywords**: ("anorexia*")) *OR* (**Keywords**: ("binge eating*")) *OR* (**Keywords**: ("bulimia")) *OR* (**Keywords**: ("disordered eating")))))
