# Supplementary material for: Non-interpersonal traumatic events in patients with eating disorders: a systematic review
Source: Front Psychol. 2024 Jun 17;15:1397952. doi: 10.3389/fpsyg.2024.1397952 (PMC11216314; doi:10.3389/fpsyg.2024.1397952)
Supplement: Supplementary file 2 [file Table_2.docx]

| **ED measurements**  Table 2 | |
| --- | --- |
| EDE | *The Eating Disorder Examination* is a structured clinical interview designed for diagnostic purposes, specifically to assess eating disorder psychopathology. It assesses the frequency and severity of eating disorder behaviors and attitudes over the past 28 days with 41 items categorized into 4 subscales; 1) Dietary Restraint, 2) Eating Concern, 3) Weight Concern and 4) Concerns regarding shape. Additionally, a shorter version, the EDE-16, comprises 16 items (Fairburn et al., 2014) |
| EDE-Q | *The Eating Disorder Examination Questionnaire,* is a self-report questionnaire, adapted from the EDE interview. It contains 28 items, which assesses eating disorder thoughts and behaviors over the past 28 days (Fairburn et al., 2008) |
| EDI | *The Eating Disorder inventory*, is a self-report questionnaire, designed to assess behavioral and psychological traits which are commonly associated with anorexia nervosa and bulimia nervosa, and is mainly used as a screening tool. It includes scales measuring the drive for thinness, body dissatisfaction, purge, and binge behaviors (Garner et al., 1983) |
| EAT-26 | *The Eating Attitudes Test*, is a self-report questionnaire consisting of 26 items rated on a 6-point Likert-scale. A cutoff score of 20 suggests a high level of concern about weight, dieting, and problematic eating behavior (Garner et al., 1982) |
| SCID | *The Structured Clinical interview for DSM Disorders*, is a widely used semi-structured interview covering the major DSM-5 Axis 1 diagnosis. Thus, section H, one of nine subsections, covers the criteria for eating disorders (First et al., 1997) |
| SEDI | *The Structured ED Interview,* is a semi-structured DSM-IV-based interview developed specifically for the Stepwise system*.* The questionnaire comprises 20–25 questions depending on which additional  questions need to be asked (De Man Lapidoth & Birgegaard, 2010) |
| ED100K | The *ED100K-v1* questionnaire is an online self-report questionnaire that operationalizes the DSM-IV criteria for all eating disorders (now adapted for DSM-5) (Thornton et al., 2018) |
| SIAB | The *Structured Interview for Anorexia Nervosa and Bulimic Syndromes*, is a semi-standardized interview assessing specific as well as *general psychopathology* (SIAB-P) and of *family interaction* (SIAB-FAM) in eating disorders (Fichter et al., 1991) |
| AUDADIS-5 | *Alcohol Use Disorder and Associated Disabilities Interview Schedule*, is a fully structured diagnostic interview designed to assess alcohol use disorders and related psychiatric conditions, according to DSM criteria in both the clinical and general population (Grant et al., 2011). |
| YBC-EDS | *Yale-Brown-Cornell Eating Disorder Scale,* is a self-report checklist, whereby the severity and types of core compulsions and obsessions specific related to eating disorders are assessed. This includes items about binge-eating episodes, food obsession, and compensatory behaviors (Sunday et al., 1995). |
| **Trauma measurements** | |
| LEC | *Life Event Checklist* is a self-reported trauma assessment tool. It lists 16 potentially traumatic events. The items assess different levels of exposure to potentially traumatic events; being a victim, a witness or being close to someone who has experienced a traumatic event (Gray et al., 2004) |
| CTQ | *Childhood Trauma Questionnaire* is a patient-rated assessment tool consisting of 70 items. The instrument includes one scale of childhood traumatic events, and another scale of recent traumatic events. The items assess both age and severity of the traumatic event (Pennebaker & Susman, 2013) |
| LYLES | *Linköbing Youth Life Experience Scale* is an inventory containing 41 questions about trauma-history. 18 items describe non-interpersonal traumatic experiences,13 items identify interpersonal traumatic experiences, and 10 involve adverse circumstances or experiences in childhood (Nilsson et al., 2010). |
| SLESQ | *Stressful Life Event Screening Questionnaire* is a 13 item self-report instrument developed to assess different types of stressful events in clinical as well as non-clinical populations (Goodman et al., 1998) |
| ETI | *Essens Trauma-Inventory* is a self-rating assessment tool consisting of 15 items that assess potentially traumatic events and 23 items that assess post-traumatic symptomatology according to DSM-IV. The inventory includes 4 subscales; intrusion, avoidance, hyperarousal and dissociation (Tagay et al., 2007). |
| PDS | *Posttraumatic Stress Symptom Scale* consists of 12 primary questions about the occurrence of a specific traumatic event. Additionally the scale includes 4 items that assess the nature of the stressor and 17 items that assess symptoms of PTSD (Foa et al.,  1997) |
| RFI | *Oxford Risk Factor Interview* measures the psychological, social and biological factors believed to be risk factors for developing an eating disorder. The interview focuses on the period prior to the onset of the clinical eating disorder or age of 18. Items of the interview assess degree and frequency of exposure (Fairburn et al., 1997) |
| SCID | *The structured clinical interview for DSM-5 disorders* is a semi-structured interview developed to assess the axis 1 diagnosis in DSM-5. Section F in this interview covers PTSD and it consists of questions about lifetime trauma exposure (Zawadzki et al., 2015) |
