# Supplementary material for: Non-interpersonal traumatic events in patients with eating disorders: a systematic review
Source: Front Psychol. 2024 Jun 17;15:1397952. doi: 10.3389/fpsyg.2024.1397952 (PMC11216314; doi:10.3389/fpsyg.2024.1397952)
Supplement: Supplementary file 4 [file Table_4.docx]

References

References in table 2

Backholm, K., Isomaa, R., & Birgegård, A. (2013). The prevalence and impact of trauma history in eating disorder patients. *European journal of psychotraumatology*, *4*(1), 22482.

Brewerton, T. D., Perlman, M. M., Gavidia, I., Suro, G., Genet, J., & Bunnell, D. W. (2020). The association of traumatic events and posttraumatic stress disorder with greater eating disorder and comorbid symptom severity in residential eating disorder treatment centers. *International Journal of Eating Disorders*, *53*(12), 2061-2066.

Convertino, A. D., Morland, L. A., & Blashill, A. J. (2022). Trauma exposure and eating disorders: Results from a United States nationally representative sample. *International journal of eating disorders*, *55*(8), 1079-1089.

Degortes, D., Santonastaso, P., Zanetti, T., Tenconi, E., Veronese, A., & Favaro, A. (2014). Stressful life events and binge eating disorder. *European Eating Disorders Review*, *22*(5), 378-382.

Groth, T., Hilsenroth, M., Boccio, D., & Gold, J. (2020). Relationship between trauma history and eating disorders in adolescents. *Journal of Child & Adolescent Trauma*, *13*, 443-453.

White, A. A. H., Pratt, K. J., & Cottrill, C. (2018). The relationship between trauma and weight status among adolescents in eating disorder treatment. *Appetite*, *129*, 62-69.

Kjaersdam Telléus, G., Lauritsen, M. B., & Rodrigo-Domingo, M. (2021). Prevalence of various traumatic events including sexual trauma in a clinical sample of patients with an eating disorder. *Frontiers in psychology*, *12*, 687452.

Lejonclou, A., Nilsson, D., & Holmqvist, R. (2014). Variants of potentially traumatizing life events in eating disorder patients. *Psychological Trauma: Theory, Research, Practice, and Policy*, *6*(6), 661.

Lie, S. Ø., Bulik, C. M., Andreassen, O. A., Rø, Ø., & Bang, L. (2021). Stressful life events among individuals with a history of eating disorders: a case-control comparison. *BMC psychiatry*, *21*, 1-12.

Longo, P., Bertorello, A., Panero, M., Abbate-Daga, G., & Marzola, E. (2019). Traumatic events and post-traumatic symptoms in anorexia nervosa. *European Journal of Psychotraumatology*, *10*(1), 1682930.

Longo, P., Marzola, E., De Bacco, C., Demarchi, M., & Abbate-Daga, G. (2020). Young patients with anorexia nervosa: the contribution of post-traumatic stress disorder and traumatic events. *Medicina*, *57*(1), 2.

Reyes-Rodríguez, M. L., Von Holle, A., Ulman, T. F., Thornton, L. M., Klump, K. L., Brandt, H., ... & Bulik, C. M. (2011). Posttraumatic stress disorder in anorexia nervosa. *Psychosomatic medicine*, *73*(6), 491-497.

Tagay, S., Schlottbohm, E., Reyes-Rodriguez, M. L., Repic, N., & Senf, W. (2014). Eating disorders, trauma, PTSD, and psychosocial resources. *Eating disorders*, *22*(1), 33-49.

Tagay, S., Schlegl, S., & Senf, W. (2010). Traumatic events, posttraumatic stress symptomatology and somatoform symptoms in eating disorder patients. *European Eating Disorders Review: The Professional Journal of the Eating Disorders Association*, *18*(2), 124-132.

Thornley, E., Vorstenbosch, V., & Frewen, P. (2016). Gender differences in perceived causal relations between trauma-related symptoms and eating disorders in online community and inpatient samples. *Traumatology*, *22*(3), 222.

Vieira, A. I., Machado, B. C., Machado, P. P., Brandão, I., Roma‐Torres, A., & Gonçalves, S. (2017). Putative risk factors for non‐suicidal self‐injury in eating disorders. *European eating disorders review*, *25*(6), 544-550.

References in table 3

de Man Lapidoth, J., & Birgegård, A. (2010). Validation of the structured eating disorder interview (SEDI) against the eating disorder examination (EDE). Stockholm: Karolinska Institutet.

Fairburn, C. G., & Beglin, S. J. (2008). Eating disorder examination questionnaire. *Cognitive behavior therapy and eating disorders, 309,* 313.

Fairburn, C. G., Cooper, Z., & O’Connor, M. (2014). Eating disorder examination (Edition 17.0 D), 265–308. <https://www.credo-oxford.com/pdfs/EDE_17.0D.pdf>

Fairburn, C. G., Cooper, Z., & O’Connor, M. (2014). Eating Disorder Examination (Edition 17.0D). Retrieved from http://www.credo-oxford.com/pdfs/EDE_17.0D.pdf.

Fairburn, C. G., Welch, S. L., Doll, H. A., Davies, B. A., & O'Connor, M. E. (1997). Risk factors for bulimia nervosa: A community-based case-control study. *Archives of General psychiatry*, *54*(6), 509-517.

Fichter, M. M., Elton, M., Engel, K., Meyer, A. E., Mall, H., & Poustka, F. (1991). Structured Interview for Anorexia and Bulimia Nervosa (SIAB): Development of a new instrument for the assessment of eating disorders. *International Journal of Eating Disorders, 10*(5), 571-592.

First, M. B., & Williams, J. B. (2017). *Structured Clinical Interview for DSM-5: Research Version (SCID-5-RV): Reference Interview:" anxious Decorator"*. Biometrics Research, Psychiatry, Columbia University at the New York State Psychiatric Institute.

Foa, E. B., Cashman, L., Jaycox, L., & Perry, K. (1997). The validation of a self-report measure of posttraumatic stress disorder: the Posttraumatic Diagnostic Scale. *Psychological assessment*, *9*(4), 445.

Garner, D. M., Olmsted, M. P., Bohr, Y., & Garfinkel, P. E. (1982). The eating attitudes test: psychometric features and clinical correlates. *Psychological medicine*, *12*(4), 871-878.

Garner, D. M., Olmstead, M. P., & Polivy, J. (1983). Development and validation of a multidimensional eating disorder inventory for anorexia nervosa and bulimia. *International journal of eating disorders*, *2*(2), 15-34.

Goodman, L. A., Corcoran, C., Turner, K., Yuan, N., & Green, B. L. (1998). Assessing traumatic event exposure: General issues and preliminary findings for the stressful life events screening questionnaire. *Journal of Traumatic Stress, 11*(3), 521-542. doi:10.1023/A:1024456713321

Grant, B. F., Goldstein, R. B., Chou, S. P., Saha, T. D., Ruan, W. J., Huang, B., ... & Hasin, D. S. (2011). The alcohol use disorder and associated disabilities interview schedule-diagnostic and statistical manual of mental disorders, version (AUDADIS-5). *Rockville, MD: National Institute on Alcohol Abuse and Alcoholism*.

Gray, M. J., Litz, B. T., Hsu, J. L., & Lombardo, T. W. (2004). Psychometric properties of the life events checklist. *Assessment (Odessa, Fla.), 11*(4), 330-341. doi:10.1177/1073191104269954

Nilsson, D., Gustafsson, P., Larsson, J., & Svedin, C. (2010). Evaluation of the linköping youth life experience scale. *The Journal of Nervous and Mental Disease, 198*(10), 768-774. doi:10.1097/NMD.0b013e3181f4acb6

Pennebaker, J. W., & Susman, J. R. (2013). Childhood trauma questionnaire. *Measurement Instrument Base for the Social Science*.

Sunday, S. R., Halmi, K. A., & Einhorn, A. (1995). The Yale‐Brown‐Cornell eating disorder scale: A new scale to assess eating disorder symptomatology. *International Journal of Eating Disorders, 18*(3), 237-245.

Tagay, S., Erim, Y., Stoelk, B., Möllering, A., Mewes, R., & Senf, W. (2007). The Essen Trauma-Inventory (ETI)—A screening instrument of identification of trau-

matic events and posttraumatic disorders. Zeitschrift für Psychotraumatologie, Psychotherapiewissenschaft, *Psychologische Medizin, 1*, 75–89.

Thornton, L. M., Munn-Chernoff, M. A., Baker, J. H., Juréus, A., Parker, R., Henders, A. K., ... & Bulik, C. M. (2018). The anorexia nervosa genetics initiative (ANGI): overview and methods. *Contemporary Clinical Trials, 74*, 61-69.

Zawadzki, B., Popiel, A., Cyniak-Cieciura, M., Jakubowska, B., & Pragłowska, E. (2015). Diagnosis of posttraumatic stress disorder (PTSD) by the structured clinical interview SCID-I. *Psychiatria Polska, 49*(1), 159-169. doi:10.12740/PP/32214
